# Aberrant *DOCK2*, *GRASP*, *HIF3A* and *PKFP* Hypermethylation has Potential as a Prognostic Biomarker for Prostate Cancer

**DOI:** 10.3390/ijms20051173

**Published:** 2019-03-07

**Authors:** Marianne T. Bjerre, Siri H. Strand, Maibritt Nørgaard, Helle Kristensen, Anne KI Rasmussen, Martin Mørck Mortensen, Jacob Fredsøe, Peter Mouritzen, Benedicte Ulhøi, Torben Ørntoft, Michael Borre, Karina D. Sørensen

**Affiliations:** 1 Department of Molecular Medicine (MOMA), Aarhus University Hospital (AUH), Palle Juul-Jensens Boulevard 99, 8200 Aarhus N, Denmark; mtbjerre@clin.au.dk (M.T.B.); siri.strand@clin.au.dk (S.H.S.); mno@clin.au.dk (M.N.); jcf@clin.au.dk (J.F.); orntoft@clin.au.dk (T.Ø.); 2 Department of Urology, Aarhus University Hospital (AUH), Palle Juul-Jensens Boulevard 99, 8200 Aarhus N, Denmark; Martin.Moerck.Mortensen@vest.rm.dk (M.M.M.); borre@clin.au.dk (M.B.); 3 Exiqon – a Qiagen company, Skelstedet 16, 2950 Vedbæk, Denmark; vejsehelle@hotmail.com (H.K.); anne.karin.rasmussen@gmail.com (A.K.R.); mouritzenpeter0@gmail.com (P.M.); 4 Department of Pathology, Aarhus University Hospital (AUH), Palle Juul-Jensens Boulevard 99, 8200 Aarhus N, Denmark; beneulho@rm.dk

**Keywords:** DNA methylation, prostate cancer, epigenetics, biomarker, diagnosis, prognosis

## Abstract

Prostate cancer (PCa) is a clinically heterogeneous disease and currently, accurate diagnostic and prognostic molecular biomarkers are lacking. This study aimed to identify novel DNA hypermethylation markers for PCa with future potential for blood-based testing. Accordingly, to search for genes specifically hypermethylated in PCa tissue samples and not in blood cells or other cancer tissue types, we performed a systematic analysis of genome-wide DNA methylation data (Infinium 450K array) available in the Marmal-aid database for 4072 malignant/normal tissue samples of various types. We identified eight top candidate markers (cg12799885, *DOCK2*, *FBXO30*, *GRASP*, *HIF3A*, *MOB3B*, *PFKP*, and *TPM4*) that were specifically hypermethylated in PCa tissue samples and hypomethylated in other benign and malignant tissue types, including in peripheral blood cells. Potential as diagnostic and prognostic biomarkers was further assessed by the quantitative methylation specific PCR (qMSP) analysis of 37 nonmalignant and 197 PCa tissue samples from an independent population. Here, all eight hypermethylated candidates showed high sensitivity (75–94%) and specificity (84–100%) for PCa. Furthermore, *DOCK2*, *GRASP*, *HIF3A* and *PKFP* hypermethylation was significantly associated with biochemical recurrence (BCR) after radical prostatectomy (RP; 197 patients), independent of the routine clinicopathological variables. *DOCK2* is the most promising single candidate marker (hazard ratio (HR) (95% confidence interval (CI)): 1.96 (1.24–3.10), adjusted *p* = 0.016; multivariate cox regression). Further validation studies are warranted and should investigate the potential value of these hypermethylation candidate markers for blood-based testing also.

## 1. Introduction

Prostate cancer (PCa) is the fourth most commonly diagnosed malignancy in the world and is the most prevalent cancer among men [1,2]. Suspicion of PCa is typically raised by an elevated serum prostate specific antigen (PSA) test and/or an abnormal digital rectal examination (DRE). This is generally followed by ten to twelve trans-rectal ultrasound-guided (TRUS) prostate biopsies, which are histologically evaluated for presence of PCa [3]. However, the PSA test is prostate- rather than PCa-specific, and up to 75% of initial TRUS-biopsy procedures are negative [4]. Conversely, up to 25% of clinically significant prostate cancers are missed at the initial TRUS-biopsy [5], together leading to many repeat biopsies and delayed detection of potentially aggressive PCa. Early intervention is crucial to prevent potentially aggressive PCa from metastatic progression, which is associated with high mortality [6], however, the majority of newly diagnosed early stage prostate cancers are clinically insignificant and do not benefit from immediate treatment. Indeed, localized PCa can be cured by radical prostatectomy (RP), while low-risk localized PCa may be safely monitored by active surveillance (AS) [7]. Currently, initial treatment choice is based exclusively on clinical variables (mainly PSA, clinical T-stage (cT), and the biopsy Gleason Score (GS) [8]), but these are suboptimal, leading to the overtreatment of many clinically insignificant prostate cancers as well as the delayed/suboptimal treatment of aggressive prostate cancers [9,10]. Hence, there is a strong clinical need for better diagnostic and prognostic biomarkers for PCa. Ideally, such novel biomarkers should be highly PCa-specific and suitable for a minimally invasive testing, e.g., in blood samples.

PCa is characterized by a relatively small mutational load in the early stages of the disease [11], whereas epigenetic changes in the DNA methylome occur frequently and consistently [12]. Hypermethylation of promoter-associated CpG islands has been closely linked with the silencing of tumor suppressor genes [13] and has shown promising diagnostic and prognostic biomarker potential for PCa in tissue-based studies [14,15,16,17], although translation into blood-based tests has been slow. Indeed, most previous studies aimed at developing blood-based DNA methylation markers for PCa have not assessed if a given candidate marker was hypermethylated in other (non-prostate) tissue types also or in peripheral blood cells [18], which could increase the risk of false positive signals in cell-free DNA (cfDNA) in blood (plasma/serum).

In the present study, we aimed to identify novel PCa-specific DNA methylation candidate markers with potential for future blood-based testing by also taking into account DNA methylation levels in other tissue types. To this end, we used a systematic discovery approach based on a very large Infinium HumanMethylation450 (450K) array dataset from the Marmal-aid database [19]. Marmal-aid contains genome-wide DNA methylome data (450K) from over 14,000 samples, representing 22 different normal/malignant human tissue types (prostate, bladder, colorectal, lung, kidney, prostate and several other organs) and more than 50 distinct cell types (e.g., several leukocyte subtypes). We discovered and validated eight candidate markers: *DOCK2* (dedicator of cytokinesis 2), cg12799885 (intergenic), *FBXO30* (F-box protein 30), *GRASP* (general receptor for the phosphoinositides 1-associated scaffold protein), *HIF3A* (hypoxia inducible factor 3 subunit alpha), *MOB3B* (MOB kinase activator 3B), *PFKP* (phosphofructokinase, platelet), and *TPM4* (tropomyosin 4). These markers were hypermethylated in PCa tissue samples and hypomethylated in normal prostate tissue, other normal and cancer tissues and in peripheral blood cells (PBC), suggesting promising diagnostic potential for PCa. Four of the candidates (*DOCK2*, *GRASP*, *HIF3A* and *PKFP*) also showed significant prognostic potential beyond routine clinicopathological parameters for the prediction of post-operative biochemical recurrence in a cohort of 197 patients who underwent radical prostatectomy. 

## 2. Results

### 2.1. Large-Scale Bioinformatics Analysis for the Discovery of PCa-Specific Candidate DNA Methylation Markers

**Figure 1:**
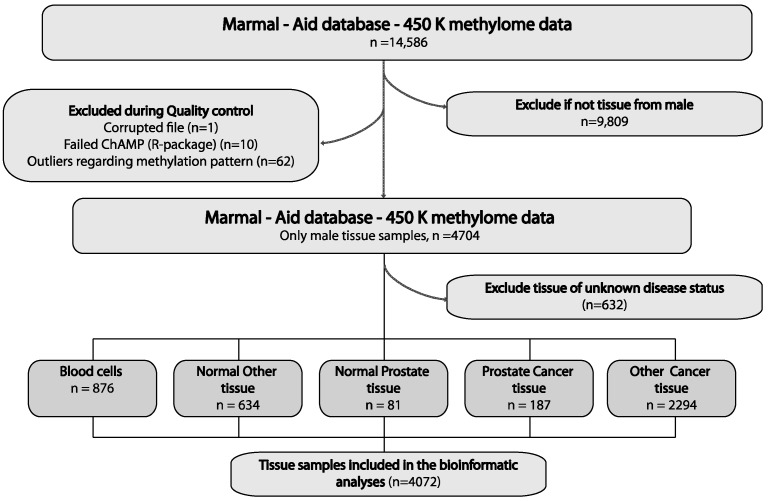
Selection of samples and biomarker candidates. Tissue sample selection for bioinformatics analyses. We selected male samples (*n* = 4072) with known disease status to ensure specificity for prostate cancer (PCa) versus other cancers and non-malignant tissues.

In order to identify novel diagnostic and prognostic biomarker candidates for PCa, we used the Illumina 450K methylation datasets from the Marmal-aid database [19]. Out of 14,486 human tissue samples with the 450K data available in the Marmal-aid database, we initially excluded 9809 samples that were not from men, 73 samples that failed quality control and 632 samples of unknown or ambiguous disease status (Figure 1). The remaining 4047 samples, all derived from men and of known tissue type and disease status, were used for further bioinformatics analysis (Table S1). Thus, the final discovery set included 450K methylome data from 81 normal prostate and 187 PCa tissue samples, 634 normal and 2294 cancer samples from other tissue types than prostate and 876 peripheral blood cell (PBC) samples (Figure 1, Table S1). The rational for also including non-prostate sample types in the discovery phase is that we aimed to identify genes specifically hypermethylated in PCa tissue samples and not in blood cells or other cancer tissue types, increasing the likelihood that these candidate markers would also be suitable for future blood-based testing for PCa.

**Figure 2:**
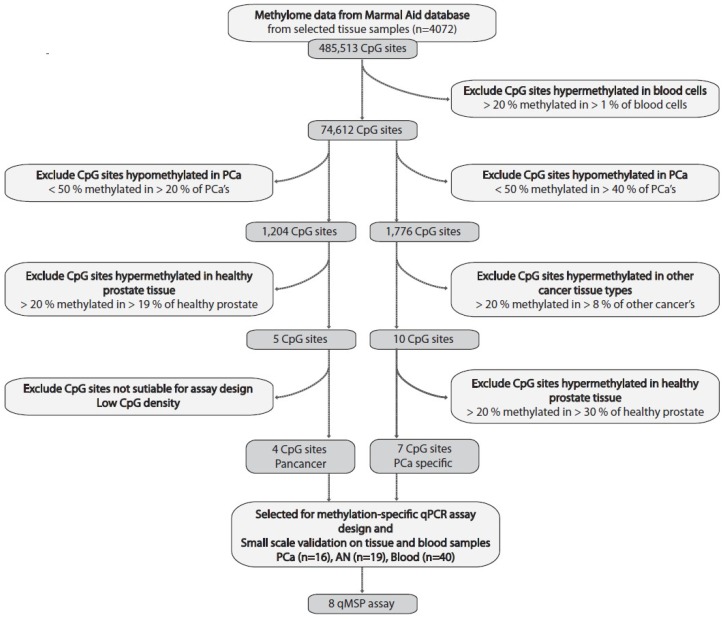
Flow chart of biomarker discovery process using 450K array data from the Marmal-aid database. Bioinformatics analyses: To identify CpG sites that may also be suitable for a future blood-based testing, we initially excluded CpG sites that displayed signs of methylation in blood cells (*n* = 876 samples). Next, we used a 2-stringed discovery approach to identify: 1) “Pan-cancer” biomarker candidates; we selected CpG sites with hypermethylation in PCa tissue samples and excluded CpG sites with hypermethylation in normal prostate tissue. 2) PCa-specific biomarker candidates; we selected CpG sites with hypermethylation in PCa tissue samples and excluded CpG sites with hypermethylation in other cancer types and/or in normal prostate tissue samples. This resulted in the selection of four “pan-cancer” and seven “PCa-specific” CpG sites suitable for the qMSP assay design.

To minimize the future risk of false positive signals in plasma, caused by methylated cfDNA derived from PBCs, we initially filtered out all of the CpG sites (*n* = 410,901 out of 485,513 CpG sites interrogated on the 450K array) that showed detectable levels of methylation (ß > 0.2) in more than 1% of the 876 PBC samples analyzed (Figure 2). Next, we used a two-stringed biomarker discovery approach based on the remaining 74,612 CpG sites (Figure 2, further details on the candidate marker selection criteria are given in Section 4.1). 

The first approach (Figure 2, left) was the least stringent and was simply aimed to identify DNA methylation marker candidates that distinguished PCa from healthy prostate tissue samples, but not necessarily distinguishing PCa from other cancer types. Although such “pan-cancer“ markers may not be useful for PCa screening in plasma due to risk of false positive signals from other malignancies, they could potentially improve specificity for PCa in men with elevated PSA. After the exclusion of 73,408 CpG sites that were hypomethylated in PCa tissue samples (ß < 0.5 in >20% of PCa samples), as well as 1119 CpG sites that were hypermethylated in normal prostate tissue samples (ß > 0.2 in >19% of samples), we identified five top candidate hypermethylation markers for PCa (Figure 2). Four of these (*DOCK2*, *HIF3A*, *GRASP* and *PFKP*) were selected for further validation, as they had a high CpG density (not shown) and hence were suitable for the design of quantitative methylation specific PCR (qMSP) assays (see Section 2.2). 

**Figure 3:**
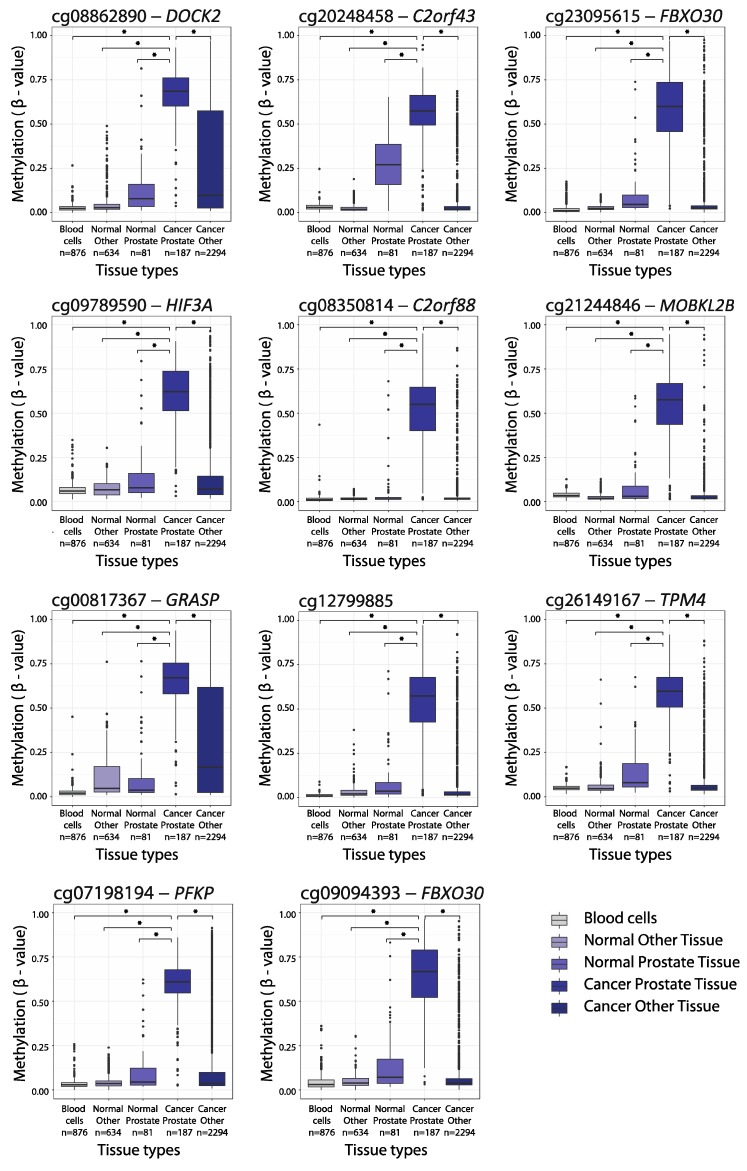
Methylation levels (ß-values) for the 11 selected biomarker candidates in the blood cell samples (*n* = 876), normal prostate tissue samples (*n* = 81), other normal tissue samples (*n* = 634), prostate cancer tissue samples (*n* = 187) and other cancer tissue samples (*n* = 2294). Other normal/cancer sample types included 14 different groups (AML (acute myeloid leukemia)/ALL (acute lymphoblastic leukemia), bladder, CNS (central nervous system), colorectal, head and neck, liver, lung, lymphoma, kidney, melanoma, pancreatic, sarcoma, stomach and thyroid). * *p* < 0.001 (Wilcoxon Mann–Whitney test). The colored boxes indicate the 25–75th percentiles and the black horizontal lines indicate the median. Top whiskers are the 3rd quartile + 1.5 interquartile range and the bottom whiskers are the 1st quartile – 1.5 interquartile range. Round dots indicate outliers.

Our second approach (Figure 2, right) was more stringent and aimed to identify “truly” PCa-specific hypermethylation candidate markers, which were hypomethylated in normal prostate tissue (ß ≤ 0.2 in ≥70% of samples) as well as and in other types of cancer tissue samples (ß ≤ 0.2 in ≥92% of samples). We excluded 72,836 CpG sites that were hypomethylated in PCa (ß < 0.5 in >40% of samples) and 1,766 CpG sites that were hypermethylated in other cancer tissue types, as well as 3 CpG sites that were hypermethylated in normal prostate tissue samples (Figure 2). This approach identified seven candidate PCa-specific hypermethylation markers (cg12799885, *C2orf43*, *C2orf88*, *FBXO30-cg23095615, FBXO30-cg09094393*, *MOB3B* and *TPM4*), all of which were suitable for the qMSP assay design and were selected for further validation (see Section 2.2 below). Of note, these candidates were also hypomethylated in other normal non-prostate tissue samples (Figure 3).

In total, using 450K data from Marmal-aid, we identified 11 candidate markers (CpG sites) that were essentially unmethylated (median ß < 0.06) in PBC samples (*n* = 876), while at the same time being significantly hypermethylated in PCa tissue (*n* = 187) when compared to normal prostate tissue samples (*n* = 81) and compared to 14 other normal/malignant tissue types (*n* = 634/*n* = 2294) (median ß difference > 0.30; *p* < 0.001, Mann–Whitney test; Figure 3). For all of the 11 CpG sites, the median methylation level (ß-value) was >0.55 in the PCa samples, and <0.27 in the non-malignant prostate specimens and <0.07/<0.17 for the other normal/malignant tissue types (Figure 3). Further details on the methylation levels for each candidate marker in individual cancer types are given in Supplementary Figure S1.

### 2.2. Independent Experimental Validation of Top Candidate Hypermethylation Markers

Next, we performed a small-scale experimental validation through the qMSP analysis of an independent set of 19 adjacent normal (AN) and 16 PCa tissue samples, as well as 40 blood (PBC) samples from male donors (Figure S2). Out of the 11 biomarker candidates analyzed, eight (cg12799885, *DOCK2*, *FBXO30-cg23095615*, *GRASP*, *HIF3A*, *MOB3B*, *PFKP* and *TPM4*) were selected for further large-scale validation (Section 2.3), as each of these showed high sensitivity (75–94%) and high specificity (84–100%) for PCa compared to AN and, furthermore, were undetectable in the PBC samples (0% false positive rate) (Table S2). 

### 2.3. Large-Scale Validation of Diagnostic Potential

**Figure 4:**
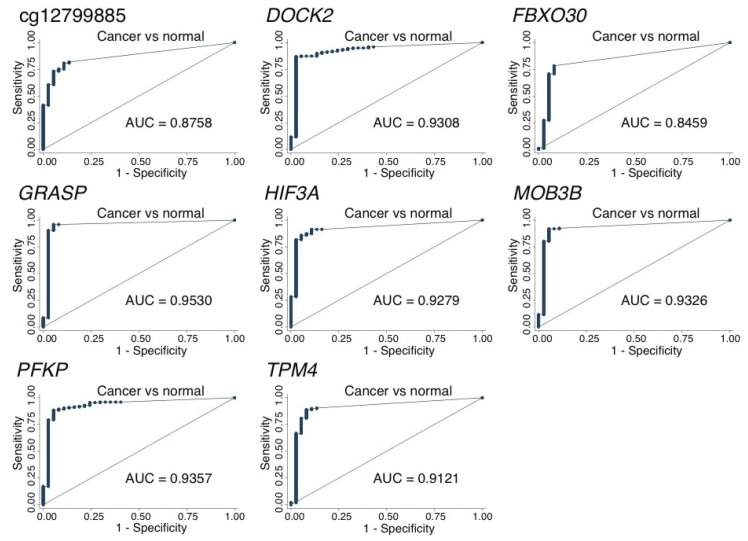
Diagnostic potential of selected methylation biomarker candidates. Receiver operating characteristics (ROC) curve analysis of the radical prostatectomy (RP) tissue samples (*n* = 197) as compared to the nonmalignant prostate tissue samples (28 AN and 9 BPH). AUC: Area under the curve.

In order to further validate the diagnostic potential of our eight selected DNA methylation marker candidates, we carried out qMSP analyses on a larger patient sample set, counting in total 197 PCa samples, 28 AN samples and 9 benign prostatic hyperplasia (BPH) tissue samples (Table 1). For all eight candidates, the PCa samples were significantly hypermethylated as compared to the non-malignant prostate tissue samples (AN/BPH) (*p* < 0.001, Mann–Whitney test; Supplementary Figure S3). By receiver operating characteristics (ROC) curve analysis, the corresponding areas under the curve (AUC) ranged from 0.85 (*FBXO30*) to 0.95 (*GRASP*) (Figure 4). Furthermore, when fixing specificity at 95%, the sensitivity for PCa ranged from 71% (*FBXO30*) to 96% (*GRASP*) (Supplementary Table S3). Thus, all eight methylation biomarker candidates (cg12799885, *DOCK2*, *FBXO30*, *GRASP*, *HIF3A*, *MOB3B*, *PFKP* and *TPM4*) displayed promising diagnostic potential for PCa. Moreover, in The Cancer Genome Atlas (TCGA) PCa patient dataset (*n* = 532 with RNAseq and 450K data available), we found a significant inverse correlation between transcriptional gene expression and the DNA methylation levels for *DOCK2*, *FBXO30*, *GRASP*, *HIF3A*, *MOB3B*, *PFKP* and *TPM4* (*p* < 0.001; rho: 0.33–0.64; Supplementary Figure S4). Furthermore, except for *DOCK2*, all of the genes were significantly downregulated in PCa (*n* = 497) compared to the AN (*n* = 35) tissue samples in the TCGA set (*p* ≤ 0.001, Wilcoxon Mann–Whitney test, Supplementary Figure S4), consistent with hypermethylation-based epigenetic gene silencing. cg12799885 is not clearly linked to a candidate gene and hence was not included in this part of the analysis.

### 2.4. Association Between DNA Methylation Levels in PCa Tumors and Clinicopathological Variables

**Figure 5:**
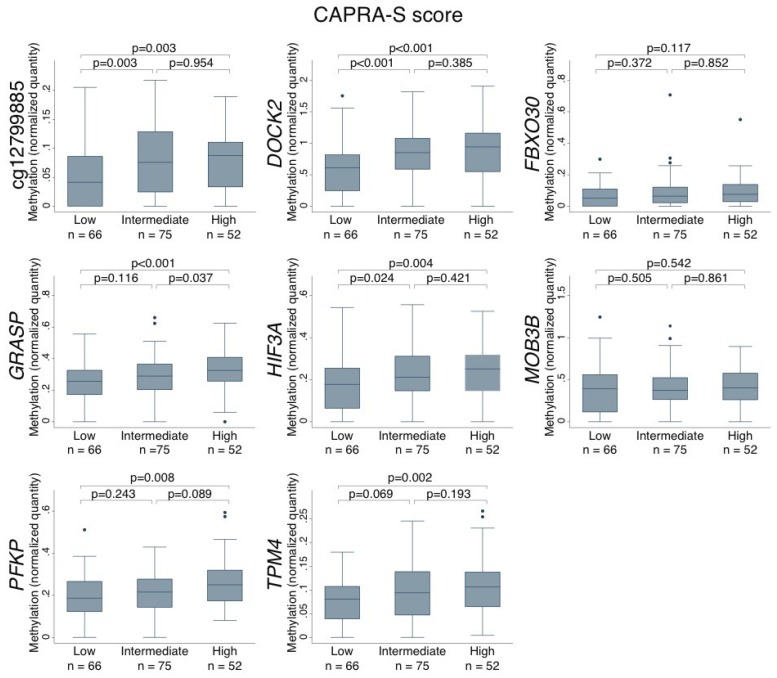
Box plots show the DNA methylation levels (normalized to *ALUC4*) for each top candidate marker as compared to the CAPRA-S risk score. CAPRA-S scores ranged from 0–2 (low), 3–5 (intermediate) and ≥6 (high). *p*-value, Wilcoxon Mann–Whitney test. The colored boxes indicate the 25–75th percentiles and the black lines indicate the median. The top whiskers are the 3rd quartile + 1.5 interquartile range and the bottom whiskers are the 1st quartile–1.5 interquartile range. Dots indicate outliers.

Next, we investigated the association between the DNA methylation levels of the eight biomarker candidates in PCa tissue samples and routinely used clinicopathological variables associated with tumor aggressiveness. We used the CAPRA-S score, which is a validated risk nomogram for biochemical recurrence (BCR) based on post-surgical clinicopathological variables and where a high score reflects adverse prognostic factors (high preoperative PSA, high prostatectomy GS, advanced pathological tumor stage (pT), positive surgical margin (SM) and/or positive lymph node involvement) [20]. For six of the candidates (cg12799885, *DOCK2*, *GRASP*, *HIF3A*, *PFKP* and *TPM4*), there was a significant correlation between high methylation levels and a high CAPRA-S score (*p* < 0.05, Wilcoxon Mann–Whitney test; Figure 5), suggesting that higher methylation levels are linked with more aggressive PCa. Consistent with this, a high methylation level was significantly associated with post-operative BCR (compared to no BCR) for the four candidates *DOCK2*, *GRASP*, *HIF3A*, and *PFKP* (adjusted *p* ≤ 0.035, Wilcoxon Mann–Whitney test.

### 2.5. Prognostic Potential in Relation to Post-Operative Biochemical Recurrence Risk

To further evaluate the prognostic potential of our eight biomarker candidates, we used univariate Cox regression analysis to investigate whether the methylation levels could predict the time to BCR after RP. Hypermethylation of *DOCK2*, *GRASP*, *HIF3A* and *PFKP* (each analyzed as a continuous variable) were all significant predictors of time to BCR after adjustment for multiple testing (Hochberg adjusted, *p* < 0.025, Table 2). Of these four candidates, *DOCK2* was the strongest single predictor of time to BCR after RP (HR (95%): 2.50 (1.59–3.94), adjusted *p* = 0.001; Table 2) in our RP cohort. All routine prognostic clinicopathological parameters (preoperative PSA, SM status, pathological GS (pGS) and pT-stage) were also significant predictors of time to BCR in the univariate Cox regression analysis (*p* < 0.026, Table 2), indicating that this is a representative RP cohort. 

**Figure 6:**
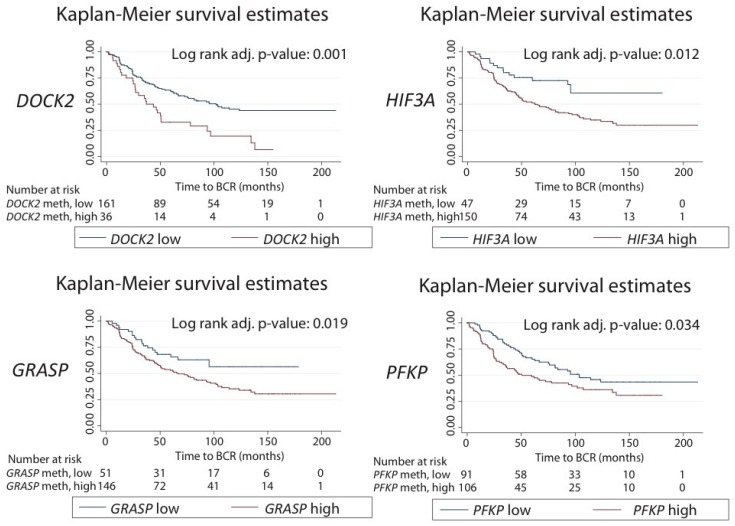
Kaplan–Meier plots with biochemical recurrence (BCR) as an endpoint in the Danish RP cohort. For each marker candidate, the patients were divided into low and high methylation subgroups based on the ROC curve analysis of the BCR status at the 36 months follow-up after RP. *p*-value from the log-rank test.

Likewise, by Kaplan–Meier analysis, we found that the median BCR-free survival time was significantly longer for patients with low methylation levels compared to those with high methylation level for each of the four candidates: *DOCK2* (101 versus 37 months, adjusted *p* = 0.001, log-rank test), *HIF3A* (>180 months versus 62 months; adjusted *p* = 0.012), *GRASP* (>178 months versus 65 months, adjusted *p* = 0.019) and *PFKP* (103 months versus 51 months; adjusted *p* = 0.034) (Figure 6). 

Furthermore, all four candidates remained significant predictors of time to BCR after adjustment for routine clinicopathological factors in the multivariate Cox regression analysis: *DOCK2* (HR (95% CI): 1.96 (1.24–3.10), adjusted *p* = 0.016), *HIF3A* (HR (95% CI): 4.73 (1.19–18.78), adjusted *p* = 0.037), *GRASP* (HR (95% CI); 5.24 (1.11–24.88), adjusted *p* = 0.037), and *PFKP* (HR (95% CI)); 6.65 (1.23–36.12)) adjusted *p* = 0.037) (Table 3). Thus, the hypermethylation of *DOCK2*, *GRASP*, *HIF3A* and *PFKP*, respectively, are significant independent predictors of time with BCR after RP, even beyond routine clinicopathological variables. 

Next, to find the optimal single/multi-marker panel, we performed stepwise backward selection using multivariate Cox regression analysis based on *DOCK2*, *GRASP*, *HIF3A* and *PFKP* and the routine clinicopathological factors preoperative PSA, surgical margin status (SM), pGS, and pT-stage. The final multivariate prognostic model consisted of *DOCK2* together with the preoperative PSA, pGS and SM status (Table S4). The addition of *DOCK2* to the multivariate model consisting of clinicopathological factors only improved the predictive accuracy (Harrell’s C-index) from 0.692 to 0.710 (Table 3 and Table S4), suggesting improved model performance and indicating particularly promising prognostic potential for *DOCK2* hypermethylation. 

**Figure 7:**
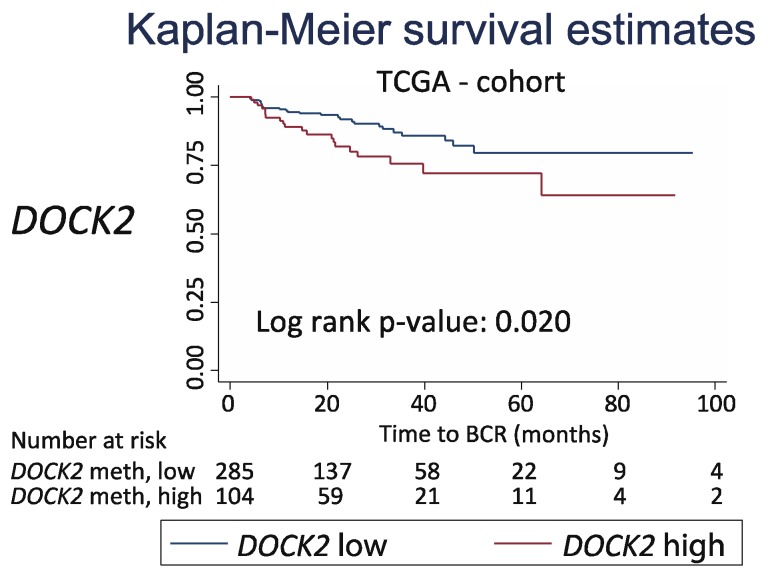
External validation of prognostic potential of *DOCK2* hypermethylation in The Cancer Genome Atlas (TCGA) cohort. Kaplan–Meyer plots with biochemical recurrence (BCR) as an endpoint. Division of patients into low and high methylation groups was based on the ROC curve analysis of the BCR status at 36 months after RP. P-value from the log-rank test.

Finally, for independent clinical validation, we tested the prognostic potential of *DOCK2* hypermethylation in another large RP patient set (450K data) available from TCGA. A high *DOCK2* methylation level was also a significant adverse predictor of BCR-free survival in this cohort (log-rank *p* = 0.020, Figure 7), thereby confirming our own findings in the Danish RP cohort. In summary, this is the first study to demonstrate and externally validate a significant prognostic value for *DOCK2* hypermethylation in relation to PCa.

### 2.6. Prognostic Potential of DOCK2 Hypermethylation in the Pre-Operative Setting

As a proof-of-principle, we assessed the possible prognostic value of *DOCK2* hypermethylation as compared to pre-operative clinicopathological factors in our large Danish PCa cohort (Table 1). We found that *DOCK2* was a significant independent predictor of time to BCR beyond the pre-operative D’Amico stratification (HR (95%): 2.26 (1.40–3.63), *p* = 0.001, multivariate Cox regression) (Table S5). Moreover, the addition of *DOCK2* to the prognostic model increased Harrell’s C-index from 0.599 (D’Amico alone) to 0.645 (D’Amico + *DOCK2*), suggesting that *DOCK2* may improve PCa risk stratification at the time of diagnosis and as a supplement to the D’Amico risk stratification.

**Figure 8:**
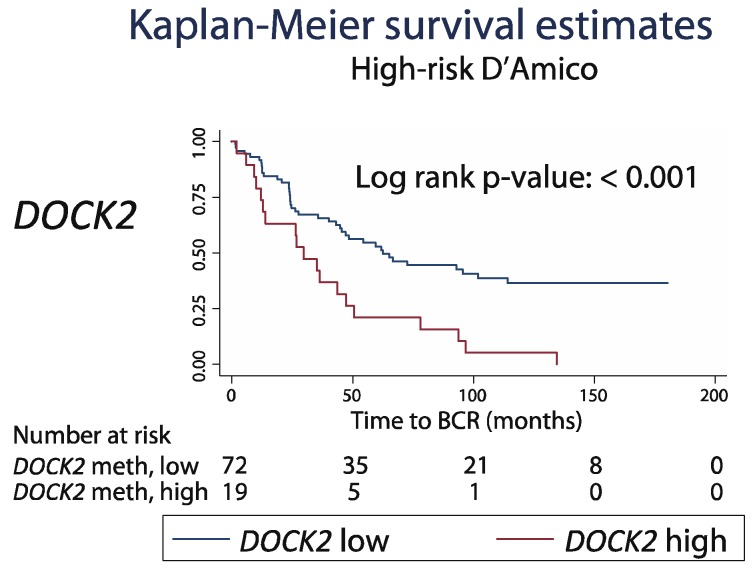
Kaplan–Meier plots with biochemical recurrence (BCR) as an endpoint in the subgroup of Danish PCa patients with D’Amico high-risk. Patients were divided into low and high methylation groups for *DOCK2* based on the ROC curve analysis of the BCR status at 36 months post-RP. *p*-value from the log-rank test.

To further address this, we tested the prognostic value of *DOCK2* hypermethylation within distinct D’Amico risk subgroups (low, intermediate and high). We found no significant added prognostic value for *DOCK2* hypermethylation in the low (*n* = 23) and intermediate risk (*n* = 80) patient subgroups (log-rank p: 0.398 and 0.174). In contrast, in the high-risk subgroup (*n* = 91), patients with high *DOCK2* methylation showed significantly shorter BCR-free survival than patients with low *DOCK2* methylation (29.8 versus 61.9 months median; Kaplan–Meier, log-rank *p* < 0.001, Figure 8). Notably, in the currently available follow-up, all patients in this sub-group with high *DOCK2* methylation experienced BCR in contrast to only 60% in the low methylation group (Figure 8), indicating that this very high-risk patient subgroup would be candidates for intensified treatment, for example, adjuvant treatment after RP. Further clinical validation is warranted to assess the possible added value of *DOCK2* methylation analyses for PCa risk stratification and to guide more personalized treatment decisions. Information on biopsy GS was not available for the cohort from TCGA, which therefore could not be included in this analysis. 

## 3. Discussion

### 3.1. Major Findings

In this study, we used the large Marmal-aid [19] database for the systematic discovery of novel PCa-specific hypermethylation biomarkers that would potentially also be suitable for future transfer into blood-based molecular tests. In summary, we identified and validated eight top candidate markers (*DOCK2*, *FBXO30*, *HIF3A*, *GRASP*, *MOB3B*, *PFKP*, *TPM4* and cg12799885) that were significantly hypermethylated in PCa compared to non-malignant prostate tissue samples, as well as to other cancer types, and which were essentially unmethylated in the PBC samples, together indicating promising diagnostic potential. Aberrant hypermethylation of *DOCK2*, *HIF3A*, *GRASP*, and *PFKP* was also a significant adverse predictor of post-operative BCR beyond the routine clinicopathological parameters, with *DOCK2* being the most promising single candidate prognostic marker. Further validation studies are needed to assess the potential future clinical value of these findings.

### 3.2. Diagnostic and Prognostic Potential of Eight Top Candidate Hypermethylation Markers

To the best of our knowledge, this is the first report to identify *DOCK2, FBXO30*, *PFKP*, and *cg12799885* as frequent targets of aberrant hypermethylation in PCa tissue samples. In addition, our results confirm and expand on earlier reports of *HIF3A*, *GRASP*, *MOB3B* and *TPM4* hypermethylation in PCa. Specifically, three previous tissue-based studies reported promising diagnostic potential for *HIF3A*, *GRASP, MOB3B* and/or *TPM4* hypermethylation in PCa with AUCs ranging from 0.93–0.99 [15,21], which is comparable to our current results (AUC range: 0.91–0.95).

Here, we also found that *DOCK2*, *FBXO30*, *HIF3A*, *GRASP*, *MOB3B*, *PFKP*, *TPM4* and cg12799885 hypermethylation was undetectable in the PBC samples (*n* = 896 samples analyzed in total by 450K/Marmal-aid or qMSP), suggesting promising future potential for blood-based testing. Accordingly, future studies should evaluate if these eight top candidate markers can be used for blood-based detection of PCa, thereby helping to reduce the number of unnecessary prostate biopsies.

Our current work is the first to demonstrate the significant prognostic value for *DOCK2*, *HIF3A*, *GRASP* and *PFKP* hypermethylation in relation to PCa. The most promising single marker in our RP cohort was *DOCK2*, which remained significant in the multivariate analysis even after adjusting for the other three candidates (see Table S3). This was also successfully validated in the large external RP cohort from TCGA. In contrast, we did not find significant independent prognostic value for *FBXO30*, *MOB3B*, *TPM4* or cg12788951 hypermethylation in our large RP cohort (*n* = 197). This is consistent with the results from our previous report [15] that also showed no significant associations between *MOB3B* hypermethylation and BCR in two distinct RP patient cohorts, as well as with another earlier case-control study that found no significant association between *TPM4* hypermethylation and PCa-specific death [22]. No previous studies have assessed the prognostic potential of *FBXO30* or cg12788951 hypermethylation in relation to PCa. 

Further clinical validation studies are warranted in order to investigate if the methylation analyses of *DOCK2, FBXO30*, *PFKP* and/or cg12799885 may be used to improve PCa risk stratification at the time of diagnosis. Indeed, from a clinical perspective, it remains a highly complicated task to decide which PCa patients to treat immediately (typically via RP or radiotherapy (RT)) and whom to observe (AS) due to a lack of accurate prognostic tools. The most commonly used nomogram for PCa risk stratification and the guidance of initial treatment decisions is the D’Amico score, but it is suboptimal and leads to both over- and under-treatment. Our current results suggest that the methylation analysis of *DOCK2* could potentially be used as an add-on to the D’Amico risk score in order to help improve the accuracy of PCa risk stratification, although further clinical validation in tissue biopsies is required to fully assess this. 

Furthermore, in the post-operative setting, several clinical nomograms for determining the risk of recurrence have been developed (e.g., Stephenson, CAPRA-S and Kattan) and can be used to identify high-risk patients eligible for adjuvant treatment and/or intensified follow-up monitoring [23]. Our results suggest that the *DOCK2*, *HIF3A*, *GRASP* and *PFKP* methylation analyses of RP specimens may be able to assist in the identification of patients at particularly high risk of BCR. Further studies are needed to investigate if the *DOCK2*, *HIF3A*, *GRASP* and/or *PFKP* methylation analysis of RP specimens may be used to help identify patients who would benefit for adjuvant compared to salvage RT [24], as well as to guiding the optimal timing of initiating androgen deprivation therapy (ADT) after recurrence [25].

### 3.3. Known Molecular Functions and Relations to Cancer.

While cg12799885 is not clearly linked to any annotated human genes, the other seven top candidate genes identified here have all been previously studied in relation to cancer, although their possible functional roles in PCa remain largely unexplored. DOCK2 is an atypical guanine nucleotide exchange factor that has been shown to be involved in the regulation of cell migration and proliferation through the small G protein Rac in various cancer types [26]. One previous study showed that siRNA-based knockdown of DOCK2 in the androgen-resistant PCa cell line PC3 had no effect on cell invasion, but inhibited CXCL13-induced cell proliferation [27]. However, the same study [27] found that the hormone-sensitive PCa cell line LNCaP did not express the DOCK2 protein, together suggesting that the function of DOCK2 in PCa may be cell-state dependent. We did not find DOCK2 expression to be significantly downregulated in PCa tissue samples as compared to adjacent normal prostate tissue samples, potentially suggesting that *DOCK2* hypermethylation may not be a driver event in early stage hormone-naïve PCa.

HIF3A belongs to the transcription factor family of hypoxia-inducible factors (HIFs), which regulate the cellular response to hypoxia [28]. While high expression of HIF3A in endometrial cancer has been correlated with poor outcomes, its functional role remains to be elucidated in relation to PCa [28]. GRASP proteins are Golgi-localized homotypic membrane tethers that organize Golgi stacks into long, continuous ribbon-like structures. Although *GRASP* is known to be hypermethylated in colorectal [29], liver [30], and prostate cancer [21], as also found in the present study, its possible functional role in relation to cancer initiation/progression remains to be examined. 

TPM4 belongs to the tropomyosin family of actin-binding proteins, which regulate the contractile system in both muscle and non-muscle cells. Similar to the downregulation of *TPM4* gene expression in PCa found in the current study, TPM4 has previously been reported as downregulated in metastatic lung cancer [31,32], but as upregulated in ovarian cancer [33]. Furthermore, in colon cancer, high expression of TPM4 has been correlated with shorter overall survival [34], while we found that *TPM4* hypermethylation was linked with transcriptional downregulation, as well as with BCR in PCa, together suggesting a cell type-specific role. 

MOB3B belongs to the MOB family of kinase regulators, which are involved in the regulation of cell division (mitotic checkpoint regulation). Here, we found *MOB3B* to be hypermethylated and downregulated in PCa. Similarly, low expression of MOB3B has been associated with malignant melanoma [35], mantle cell lymphoma [35], poor outcome of renal cancer [36] and adverse clinicopathological factors in PCa [37], together suggesting a possible tumor suppressor role for MOB3B. The FBXO30 protein is involved in protein modifications trough the ubiquitin proteasome pathway [38], but it has not previously been investigated in relation to PCa. Although we found FBXO30 to be significantly downregulated in PCa versus nonmalignant PCa tissue samples in the cohort from TCGA, others have reported FBXO30 as upregulated in PCa and for example in nasopharyngeal cancer [30]. Thus, further studies are needed to investigate the role of FBXO30 in PCa. PFKP is a member of the phosphofructokinase A protein family, which is involved in glycolysis [39]. In this study, the *PFKP* gene was found to be hypermethylated and downregulated in PCa. This is in agreement with a previous study that also reported PFKP to be downregulated in PCa [40], but in contrast to lung cancer where PFKP has been suggested as an oncogene [41]. 

In summary, further studies are needed to investigate the possible functional role of *DOCK2*, *FBXO30*, *HIF3A*, *GRASP*, *MOB3B*, *PFKP* and *TPM4* in relation to PCa. Future studies are also needed to identify possible candidate genes that could be linked to the hypermethylation of cg12799885.

### 3.4. Study Limitations 

There are some potential limitations to the present study. First, all DNA methylation analyses were limited to tissue samples, although our ultimate future goal is to develop a blood-based DNA methylation test for PCa. Thus, although blood-based analyses are considered to be beyond the scope of the present report, the next future step should be to test our top candidate PCa hypermethylation markers in the analysis of ctDNA in plasma samples. Aberrantly methylated tumor DNA is not necessarily released in sufficient amounts and may not be preserved in plasma, where only nucleosome-associated DNA fragments are protected from DNase activity [42]. Together, these factors may limit sensitivity for PCa detection by ctDNA analysis, but could potentially be mitigated by analyzing several DNA methylation candidate markers in parallel and/or very large volumes of plasma. Furthermore, to ensure the high specificity of future ctDNA-based methylation tests, we used a very stringent biomarker discovery approach that should eliminate (or at least minimize the risk of) false positive signals in the plasma from leukocyte-derived genomic DNA, which constitutes the vast majority of cfDNA, particularly in patients with early stage cancer and with relatively low tumor burden [43].

Moreover, the present study was based on available RP specimens and retrospective data collection, which may potentially lead to selection bias. However, our RP cohort used for the qMSP analyses seemed to be representative, as all of the routine clinicopathological factors (preoperative PSA, pT-stage, pGS, SM status) were significant predictors of BCR in the univariate analyses. Furthermore, we also successfully validated the prognostic potential of our top candidate prognostic marker *DOCK2* in the large external RP patient cohort available from TCGA.

Another potential limitation is the use of BCR as the clinical endpoint, since not at all RP patients with BCR will eventually develop metastatic disease or die from PCa in their lifespan [44]. We did not have a sufficient number of events to analyze these additional clinical endpoints in our RP patient cohort, nor in the external validation cohort from TCGA. Thus, further studies are needed to determine the true clinical value of *DOCK2*, *HIF3A*, *GRASP* and/or *PFKP* methylation levels for the prediction of metastatic progression and PCa-specific survival. However, given the slow progressing nature of early stage PCa, such future studies would require >10 years of clinical follow-up [45].

## 4. Materials and Methods

### 4.1. Biomarker Discovery

The methylome data (ß-value, disease status and gender) was downloaded from the Marmal-aid database [19], which contains genome-wide methylome data (Illumina InfiniumHumanMethylation450 (450K) array) from >14,000 tissue samples of various organ/cell types. The data were adjusted for intrasample variation by beta-mixture quantile (BMIQ) normalization, using the ChAMP package (version 1.8.0) for R prior to further analysis [46]. In the bioinformatic analysis, we initially excluded CpG sites that displayed signs of even low DNA methylation levels (ß-value > 0.2) in more than 1% of blood specimens to find potential biomarkers suitable for future blood-based tests. We then used a two-stringed approach to find CpG sites that had a different methylation pattern in PCa compared to normal prostate tissue samples. For search string 1 (“pan-cancer” biomarker candidates), we selected CpG sites displaying hypermethylation (ß-value > 0.5) in more than 80% of PCa tissue samples and excluded CpG sites with a methylation level where the ß-value was >0.2 in more than 19% of normal prostate tissue samples. In search string 2 (PCa-specific biomarker candidates), we selected CpG sites displaying hypermethylation (ß-value > 0.5) in more than 60% of PCa tissue samples and excluded CpG sites which were hypermethylated (ß-value > 0.2) in more than 8% of tissue samples from other cancer types as well as CpG sites, where there was a detectable methylation level (ß-value > 0.2) in more than 30% of normal prostate tissue samples.

For each top candidate gene identified, methylation-specific assays for qPCR analysis were designed using Beacon Designer^TM^ (Premier Biosoft, Palo Alto, CA, USA; Table S6). As reference genes for DNA quantification and quality control, *ALUC4* and *MYOD1* were used [14,15]. Locked nucleic acid (LMA^TM^)-based qMSP assays were designed by Exiqon—a Quiagen company (Vedbæk, Denmark). During the assay design, the LNA™ Oligo Optimizer tool (found at https://www.exiqon.com/ls/Pages/ExiqonOligoOptimizerTool.aspx) was used to ensure LNA™ oligo designs with a self-complementarity and secondary structure score below 40. The annealing temperatures (Tm) of the LNA™-enhanced oligos were predicted using the LNA™ Oligo Tm Prediction tool (found at https://www.exiqon.com/ls/Pages/ExiqonTMPredictionTool.aspx). The primers were designed to have a Tm = 59–61 °C and a probe Tm = primer Tm + 5–10 °C. The primers were purchased from Integrated DNA Technologies (Leuven, Belgium) and the Taqman^®^ probes were ordered from LGC Biosearch Technologies (Risskov, Denmark).

### 4.2. Patients and Tissue Samples

All patients provided written informed consent prior to inclusion in the study and the study was approved by The Committee on Health Research Ethics for the Central Denmark Region (No. 2000/0299, 28 March 2017) and The Danish Data Protection Agency (No. 2013-41-2041, 27 January 2016).

#### 4.2.1. Patient Samples for Small-Scale Experimental Validation

Twenty PCa tissue samples (RP specimens from patients without BCR), 20 BPH/AN, 20 whole-blood samples from PCa patients and 20 buffycoat samples (i.e., mainly leukocytes) from healthy male blood donors were used for experimental validation of the top candidate markers identified from the analysis of the Marmal-aid dataset. All tissue samples were collected at Aarhus University Hospital, Denmark. Five samples (4 PCa and 1 AN) failed the quality control (less than two out of three replicates with detectable levels of the reference genes *ALUC4* and *MYOD1*) and hence were not included in the final analyses [14,15].

#### 4.2.2. Clinical Cohort for Large-Scale Validation

Patients with histologically confirmed clinically localized PCa that were treated by curatively intended RP at Aarhus University Hospital from 1999–2013 were used for large-scale validation. The inclusion criteria were as follows: Available formalin-fixed paraffin embedded (FFPE) tissue (PCa and/or AN) which had been subjected to histological verification by an expert uropathologist, and good DNA quality (minimum DNA concentration of 100 ng/ul after DNA purification). A total of 264 PCa patients (254 PCa tissue and 37 AN tissue samples and 27 patients with matched PCa and AN tissue samples) were included in this phase of the study. As additional non-cancer controls, tissue samples from nine patients with BPH, who underwent transurethral resection of the prostate (TURP) were also included. A total of 55 PCa and 9 AN samples did not pass the final quality control during qMSP (less than two out of three replicates with detectable levels of the reference genes *ALUC4* and *MYOD1*), thus, the final cohort consisted of 197 PCa and 37 non-malignant prostate tissue samples (28 AN and 9 BPH). See Table 1 for an overview of the clinicopathological characteristics.

### 4.3. DNA Extraction, Bisulfite Conversion and Quantitative Methylation-Specific PCR Analysis (qMSP)

Genomic DNA was extracted from FFPE punch biopsies from the RP specimens (PCa and AN) or TURP specimens (BPH), as previously described [14,15,16]. Genomic DNA from blood cells was extracted from whole blood and/or buffycoat from healthy blood donors using the QIASymphony DSP DNA Midi Kit (Qiagen, Hilden, Germany, Cat#937255).

The EZ Methylation-Direct Kit (Zymo Research, Irvine, CA, USA) was used for bisulfite conversion of 1000 ng of DNA input from each sample. Next, quantitative methylation-specific PCR (qMSP) analyses were run, as previously described [14,15]. In brief, all reactions were run in triplicates with 5 ng bisulfite converted DNA as the input, using the Taqman universal mastermix (no UNG) on the Thermofisher ViiA^TM^ 7 real-time PCR system. Each 384-well plate included serially diluted methylated DNA samples for standard curve analyses, fully-methylated positive control (bisulfite converted CpGenome Universal Methylated DNA; Merck/Millipore), fully unmethylated negative control (whole-genome amplified DNA (WGA), and a no-template control (water). As reference genes for DNA quantification and quality control, *ALUC4* and *MYOD1* were used, and the data were normalized to *ALUC4* as previously described [14,15]. The quantity was arbitrarily set to 0 for patient samples with a C_T_ value >40 (undetermined) and/or a C_T_ value above the C_T_ value for the negative controls (WGA and water) in the same qMSP run, except in the small-scale experimental validation, where the quantity was set to 0 for all patient samples with a C_T_ value >37. The quality control steps for all runs encompassed the exclusion of samples with a quantification cycle (C_T_) >24 for *ALUC4*, and/or without a *MYOD1* positive signal and/or samples with <2 of the triplicate reactions amplified (after the removal of outliers >2 C_T_s lower/higher, compared to other C_T_s in triplicate).

### 4.4. TCGA Data Used for External Validation of DOCK2 Prognostic Potential

The DNA methylome data (450K array data), RNA sequencing data and clinical data for PCa tissue samples from 498 RP patients and 52 matched AN samples was downloaded from the TCGA platform [42] and used for external validation.

### 4.5. Statistical Analyses

STATA v.15 (StataCorp, Collage Station, TX, USA) and R version 3.5.0 were used for the statistical analyses [47].

Statistical significance was set at *p* < 0.05. When appropriate, the Benjamini–Hochberg method [48] was used to correct for multiple testing. The diagnostic value was assessed using the Wilcoxon Mann–Whitney test and ROC curve analyses of the methylation levels in malignant (PCa) versus non-malignant (AN/BPH) specimens. The prognostic potential of the methylation biomarker candidates was evaluated using uni- and multivariate Cox regression analyses, Kaplan–Meier analysis and two-sided log-rank tests. For the Kaplan–Meier analyses and log-rank tests, the patients were divided into high- and low-methylation groups based on cutoffs from the ROC analyses (BCR status at 36 months follow-up) for each candidate marker. Post-operative BCR (defined as PSA ≥ 0.2 ng/mL) was the clinical endpoint for all prognostic analyses. Lymph node status was not included in the multivariate analyses as very few patients (2.5%) were lymph node positive. Harrell’s C-index [49] was used to estimate predictive accuracy.

## 5. Conclusions

In this study, we identified and validated *DOCK2*, *FBXO30*, *HIF3A*, *GRASP*, *MOB3B*, *PFKP*, *TPM4* and cg12799885 as novel PCa-specific hypermethylation candidate markers. Our results also suggest promising potential for all of these candidate markers in future blood-based testing, as none of them had detectable DNA methylation levels in PBC samples. Four of the top candidate markers (*DOCK2*, *GRASP*, *HIF3A* and *PKFP*) showed prognostic potential for the prediction of BCR after RP, with the most promising single candidate marker being *DOCK2*. Indeed, we identified *DOCK2* hypermethylation as a novel independent predictor of post-operative BCR beyond routine clinicopathological parameters (pre-operative as well as post-operative) and successfully validated this in a large independent RP cohort which was available from TCGA. Further studies are warranted to assess the possible future clinical utility of this novel set of epigenetic diagnostic and prognostic biomarker candidates for PCa.

## Figures and Tables

**Table 1:** Clinicopathological characteristics of radical prostatectomy (RP) patient cohort and controls.

RP Cohort		PCa
N		197
Age years, median (range)		64 (49–77)
Preoperative PSA		
PSA ng/mL, median (range)		12.9 (2.1–61.0)
D’Amico Risk classification		
Low risk, N (%)		23 (11.7)
Intermediate risk, N (%)		80 (40.6)
High risk, N (%)		91 (46.2)
Unknown, N (%)		3 (1.5)
Pathological Gleason score		
<7, N (%)		63 (32.0)
=7, N (%)		98 (49.7)
>7, N (%)		36 (18.3)
Pathological T-stage		
<pT2c, N (%)		42 (21.3)
=pT2c, N (%)		85 (43.1)
>pT2c, N (%)		70 (35.5)
Surgical margin status		
Negative, N (%)		137 (69.5)
Positive, N (%)		60 (30.5)
Lymph node status		
Positive N1, N (%)		5 (2.5)
Negative N0, N (%)		164 (83.2)
Unknown NX, N (%)		28 (14.2)
Follow-up		
Follow-up months, median (range)		128 (7–219)
PSA recurrence, N (%)		107 (54)
**Controls**	**AN**	**BPH**
N	28	9
Age years, median (range)	64 (56–73)	70 (57–81)

AN: Adjacent normal. BPH: Benign prostatic hyperplasia. PCa: Prostate cancer. PSA: Prostate specific antigen.

**Table 2:** Univariate Cox regression analysis using biochemical recurrence (BCR) after RP as endpoint. The Danish RP cohort included 234 patients in total. The methylation biomarker candidates were analyzed as continuous variables.

Variable	Univariate Cox Regression			
	HR (95% CI)	*p*-value	Adjusted *p*-value	C-Index
cg12799885	34.73 (1.57–769.04)	0.025*	0.100	0.570
*DOCK2*	2.50 (1.59–3.94)	<0.001*	0.001*	0.615
*FBXO30*	7.52 (0.78–72.42)	0.081	0.162	0.528
*GRASP*	13.87 (3.16–60.80)	<0.001*	0.003*	0.606
*HIF3A*	6.46 (1.77–23.56)	0.005*	0.024*	0.587
*MOBKL2B*	1.22 (0.59–2.52)	0.599	0.599	0.510
*PFKP*	14.63 (2.95–72.50)	0.001*	0.006*	0.598
*TPM4*	40.03 (1.21–1317.12)	0.038*	0.114	0.547
Surgical margin (negative vs. positive)	3.24 (2.20–4.76)	<0.001*	<0.001*	0.635
Pre-op PSA dichotomized (< 10 vs ≥ 10)	1.99 (1.25–3.15)	0.004*	0.004*	0.572
Pathological GS (6-7 vs. 8-10)	2.33 (1.51–3.59)	<0.001*	<0.001*	0.56
Pathological T-stage (pT2a-T2b vs. pT2c-pT4)	1.80 (1.07–3.03)	0.026*	0.026*	0.552
Pathological N-stage	2.83 (1.13–7.04)	0.026*	0.026*	0.514
Age at diagnosis	0.96 (0.93–1.00)	0.054	0.054	0.554

GS: Gleason score. Pre-op: Preoperative. HR: Hazard ratio. Adjusted *p*-value (Hochberg corrected). C-index (Harrell’s C-index). * Significant *p*-values (<0.05).

**Table 3:** Multivariate Cox regression analysis with biochemical recurrence (BCR) as an endpoint. The Danish RP cohort included 234 patients in total.

**Variable**	**Multivariate Cox Regression**		**Final Multivariate Cox Regression**				
	HR (95% CI)	*p*-value	HR (95% CI)	*p*-value	Adj *p*-value	C-index^a^	C-index^b^
*DOCK2*-continuous	1.95 (1.20–3.17)	0.004*	1.96 (1.24–3.10)	0.004*	0.016*	0.719^a^	-
Surgical margin (negative vs. positive)	2.48 (1.60–3.84)	<0.001*	2.53 (1.70–3.75)	<0.001*	<0.001*		0.692^b^
Preoperative PSA (<10 vs ≥10)	1.82 (1.14–2.90)	0.012*	1.82 (1.14–2.89)	0.012*	0.012*		
Path. Gleason score (6–7 vs. 8–10)	1.69 (1.09–2.64)	0.020*	1.69 (1.09–2.64)	0.019*	0.019*		
Path. T-stage (pT2a-T2b vs. pT2c-pT4)	0.96 (0.54–1.70)	0.880	-	-	-	-	-
**Variable**	**Multivariate Cox Regression**		**Final Multivariate Cox Regression**				
	HR (95% CI)	*p*-value	HR (95% CI)	*p*-value	Adj *p*-value	C-index^a^	C-index^b^
*HIF3A*-continuous	4.73 (1.14–19.5)	0.032*	4.73 (1.19–18.78)	0.027*	0.037*	0.713^a^	-
Surgical margin (negative vs. positive)	2.58 (1.67–3.99)	< 0.001*	2.69 (1.81–3.98)	<0.001*	<0.001*		0.692^b^
Preoperative PSA (<10 vs ≥10)	1.82 (1.14–2.90)	0.012*	1.83 (1.15–2.92)	0.011*	0.011*		
Path. Gleason score (6–7 vs. 8–10)	1.69 (1.08–2.65)	0.022*	1.70 (1.09–2.66)	0.019*	0.019*		
Path. T-stage (pT2a-T2b vs. pT2c-pT4)	0.99 (0.56–1.76)	0.979	-	-	-	-	-
**Variable**	**Multivariate Cox Regression**		**Final Multivariate Cox Regression**				
	HR (95% CI)	*p*-value	HR (95% CI)	*p*-value	Adj *p*-value	C-index^a^	C-index^b^
*GRASP*-continuous	5.21 (1.04–26.0)	0.044*	5.24 (1.11–24.9)	0.037*	0.037*	0.708 ^a^	-
Surgical margin (negative vs. positive)	2.39 (1.54–3.71)	< 0.001*	2.53 (1.69–3.77)	<0.001*	<0.001*		0.692^b^
Preoperative PSA (<10 vs ≥10)	1.75 (1.10–2.78)	0.019*	1.74 (1.10–2.77)	0.019*	0.019*		
Path. Gleason score (6–7 vs. 8–10)	1.70 (1.09–2.67)	0.018*	1.71 (1.10–2.67)	0.017*	0.017*		
Path T-stage (pT2a-T2b vs. pT2c-pT4)	1.03 (0.58–1.81)	0.931	-	-	-	-	-
**Variable**	**Multivariate Cox Regression**		**Final Multivariate Cox Regression**				
	HR (95% CI)	*p*-value	HR (95% CI)	*p*-value	Adj *p*-value	C-index^a^	C-index^b^
*PFKP*-continuous	7.47 (1.27–43.9)	0.026*	6.65 (1.23–36.1)	0.028*	0.037*	0.710^a^	-
Surgical margin (negative vs. positive)	2.44 (1.58–3.79)	< 0.001*	2.60 (1.75–3.86)	<0.001*	<0.001*		0.692^b^
Preoperative PSA (<10 vs ≥10)	1.84 (1.15–2.96)	0.012*	1.85 (1.16–2.97)	0.010*	0.010*		
Path.Gleason score (6-7 vs. 8-10)	1.62 (1.03–2.56)	0.033*	1.65 (1.05–2.59)	0.030*	0.030*		
Path. T-stage (pT2a-T2b vs. pT2c-pT4)	1.01 (0.57–1.79)	0.977	-	-	-	-	-

Path.: Pathological. HR: Hazard ratio. Adj *p*-value: Hochberg corrected *p*-value. C-index^a^: Harrell’s C-index for the model including all variables significant in the multivariate analyses. C-index^b^: Harrell’s C-index for the model only including the clinicopathological variables significant in multivariate analyses. * Significant *p*-values (<0.05). - Not applicable.

## Supplementary Materials

Supplementary materials can be found at https://www.mdpi.com/1422-0067/20/5/1173/s1. Figure S1. Cancer type related methylation levels (ß-value) of the 11 selected biomarker candidates. Figure S2. Methylation level of biomarker candidates in AN/BPH and PCa tissue samples—small scale validation. Figure S3. Methylation levels of biomarker candidates in AN/BPH and PCa tissue samples—large scale validation. Figure S4. Correlation between RNA expression and DNA methylation for the biomarker candidates—validation in the cohort from TCGA. Table S1. Marmal-aid dataset (450K array data). Table S2. Small scale validation. Table S3. Large scale validation. Table S4. Stepwise backward selection multivariate Cox regression analyses of BCR-free survival after RP. Table S5. Multivariate Cox regression analysis of BCR-free survival in relation to the known preoperative D’Amico risk classification. Table S6. Primer and probe sequences for the qMSP assays used for small scale validation.

## Abbreviations

450K	Illumina 450K DNA methylation array
ADT	Androgen deprivation therapy
AN	Adjacent normal
AUC	Area under the curve
BCR	Biochemical recurrence
BPH	Benign prostatic hyperplasia
cfDNA	Cell-free DNA
CI	Confidence interval
C_T_	Cycle threshold
cT	Clinical T-stage
ctDNA	Circulating tumor DNA
DRE	Digital rectal examination
FFPE	Formalin-fixed paraffin embedded
HR	Hazard ratio
GS	Gleason Score
n	Number
N	N-stage
PBC	Peripheral blood cells
PCa	Prostate cancer
PSA	Prostate specific antigen
pT	Pathological T-stage
qMSP	Quantitative methylation specific PCR
qPCR	Quantitative PCR
RNAseq	RNA sequencing
ROC	Receiver operating characteristics
RP	Radical prostatectomy
RT	Radiotherapy
SM	Surgical margin
T-stage	Tumor stage
TCGA	The Cancer Genome Atlas
TRUS	Trans-rectal ultrasound
TURP	Transurethral resection of the prostate
WGA	Whole-genome amplified DNA
