# Supplementary material for: Aberrant DOCK2, GRASP, HIF3A and PKFP Hypermethylation has Potential as a Prognostic Biomarker for Prostate Cancer
Source: Int J Mol Sci. 2019 Mar 7;20(5):1173. doi: 10.3390/ijms20051173 (PMC6429171; doi:10.3390/ijms20051173)
Supplement: Supplementary file 1 [file ijms-20-01173-s001.pdf]

Table S1. Marmal-aid dataset (450K array data).

Tissue type	Disease status	Mean age	Dataset
**Prostate cancer (n = 187)**	**Cancer**	**60**	TCGA (n=176), GSE38240 (n=3), GSE55598 (n=8)
**Other cancer (n=2290)**	**Cancer**	**59**	-
Alveolar (n = 2)	Cancer	60	GSE41114 (n=2)
Bladder (n = 85)	Cancer	67	TCGA (n=85)
Blood (n = 139)	Cancer	56	TCGA (n=105), GSE39141 (n=15), GSE38235 (n=19)
Bone (n = 40)	Cancer	62	TCGA (n=10), GSE40853 (n=30)
Bone Marrow (n = 18)	Cancer	56	GSE40870 (n=18)
Brain (n = 220)	Cancer	44	TCGA (n=67), GSE36278 (n=71), GSE48462 (n=40), GSE49576 (n=1), GSE50774 (n=15), GSE50774 (n=15), GSE61160 (n=26)
Buccal (n = 2)	Cancer	60	GSE41114 (n=2)
CNS (n = 114)	Cancer	44	TCGA (n=114)
Colon (n = 151)	Cancer	65	TCGA (N=140), GSE48684 (n=11)
FOM (n = 6)	Cancer	60	GSE41114 (n=6)
Head/Neck (n = 222)	Cancer	60	TCGA (n=222)
Kidney (n = 244)	Cancer	61	TCGA (n=244)
Liver (n = 155)	Cancer	59	TCGA (n=43), GSE43273 (n=12), GSE43273 (n=100)
Lung (n = 532)	Cancer	66	TCGA (n=278), GSE39279 (n=254)
Lymphoma (n = 15)	Cancer	54	GSE42372 (n=15)
Pancreas (n = 2)	Cancer	65	GSE49149 (n=2)
Rectum (n = 54)	Cancer	65	TCGA (n=54)
Skin (n = 113)	Cancer	56	TCGA (n=113)
Stomach (n = 82)	Cancer	66	TCGA (n=82)
Thyroid Gland (n = 78)	Cancer	48	TCGA (n=78)
Tongue (n = 20)	Cancer	60	GSE41114 (n=20)
**Prostate normal (n = 81)**	**Normal**	**62**	TCGA (n=81)
**Blood (n = 876)**	**Normal**	**56**	GSE41169 (n=66), GSE42865 (n=3), GSE32148 (n=8), GSE35069 (n=60), GSE37966 (n=1), GSE39141 (n=2), GSE40005 (n=6), GSE40279 (n=317), GSE36369 (n=8), GSE43976 (n=13), GSE41782 (n=3), GSE44798 (n=5), GSE49667 (n=10), GSE51032 (n=84), GSE51388 (n=36), GSE53193 (n=6), GSE53740 (n=130), GSE54399 (n=14), GSE54643 (n=20), GSE54939 (n=2), GSE56553 (n=42), GSE51245 (n=40)
**Normal other (n=633)**	**Normal**	**59**	-
Adrenal Medulla (n = 2)	Normal	NA	GSE43298 (n=2)
Bladder (n = 10)	Normal	65	TCGA (n=10)
Bone Marrow (n = 2)	Normal	47	GSE51759 (n=2)
Brain (n = 118)	Normal	42	TCGA (n=1), GSE41826(n=42), GSE50853 (n=24), GSE53162 (n=18), GSE40360 (n=12), GSE53924 (n=20), GSE44684 (n=1)

Buccal (n = 3)	Normal	36	GSE50586 (n=3)
CNS (n = 2)	Normal	43	TCGA (n=2)
Colon (n = 24)	Normal	70	TCGA (n=21), GSE48684(3)
Head/Neck (n = 38)	Normal	62	TCGA (n=38)
Kidney (n = 136)	Normal	62	TCGA (n=136)
Liver (n = 34)	Normal	62	TCGA (n=25), GSE48325 (n=8), GSE52578 (n=1)
Lung (n = 254)	Normal	65	TCGA (n=46), GSE52401 (n=208)
Pancreas (n = 1)	Normal	NA	GSE52578 (n=1)
Rectum (n = 3)	Normal	68	TCGA (n=3)
Sperm (n = 2)	Normal	NA	TCGA (n=2)
Thyroid Gland (n = 5)	Normal	53	TCGA (n=5)

Table S2. Small scale validation.

Variable	Sensitivity	Specificity	Negative Predictive Value	Positive Predictive Value	False Positive Rate for blood cells
DOCK2	**0,94**	**1,00**	**0,95**	**1,00**	0,00
GRASP	**0,94**	**1,00**	**0,95**	**1,00**	0,00
HIF3A	**0,88**	**1,00**	**0,90**	**1,00**	0,00
FBXO30_cg23095615	**0,75**	**0,95**	**0,82**	**0,92**	0,00
MOBKL2B	**0,75**	**0,95**	**0,82**	**0,92**	0,00
PFKP	**0,81**	**0,95**	**0,86**	**0,93**	0,00
TPM4	**0,81**	**0,89**	**0,85**	**0,87**	0,00
**cg12779885**	**0,75**	**0,84**	**0,80**	**0,80**	0,00
C1orf88	0,94	0,95	0,95	0,94	0,03
C1orf43	0,94	0,05	0,50	0,45	0,03
FBXO30_cg09094393	0,88	0,89	0,89	0,88	0,05

Selected biomarker candidates are highlighted in bold.

Table S3. Large scale validation.

Gene	AUC	Sensitivity	Specificity (fixed)
cg12799885	0.876	73.2 %	94.6 %
DOCK2	0.931	87.4 %	94.6 %
FBXO30	0.846	70.7 %	94.6 %
GRASP	0.953	96.0 %	94.6 %
HIF3A	0.928	85.7 %	94.6 %
MOBKL2B	0.933	91.9 %	94.6 %
PFKP	0.936	88.4 %	94.6 %
TPM4	0.912	80.8 %	94.6 %

AUC, Area under curve in ROC curve analysis (PCa vs AN/BPH)

Table S4. Stepwise backward selection multivariant cox regression analyses of BCR-free survival after radical prostatectomy.

Variable	Multivariate cox regression		Final multivariate cox regression				
	HR (95% CI)	P-value	HR (95% CI)	P-value	Adj P-value	C-index[a]	C-index[b]
HIF3A - continuous	1.59 (0.23-10.78)	0.637	-	-	-	-	-
GRASP - continuous	1.19 (0.13-11.22)	0.879	-	-	-	-	-
PFKP - continuous	1.08 (0.08-14.93)	0.952	-	-	-	-	-
DOCK2 - continuous	1.73 (0.86-3.45)	0.122	1.96 (1.24-3.10)	0.004*	0.016*	0.719[a]	-
Pathological Gleason score (6-7 vs. 8-10)	1.67 (1.07-2.63)	0.025*	1.69 (1.09-2.64)	0.019*	0.019*		0.692[b]
Preoperative PSA dichotomized (< 10 vs >= 10)	1.85 (1.15-2.97)	0.011*	1.82 (1.14-2.89)	0.012*	0.012*		
Surgical margin (negative vs. positive)	2.58 (1.69-3.94)	<0.001*	2.53 (1.70-3.75)	<0.001*	<0.001*		
Pathological T-stage (pT2a-T2b vs. pT2c-pT4)	0.93 (0.52-1.64)	0.792	-	-	-	-	-

HR, Hazard ratio; CI, confidence interval. Adj P-value, Hochberg corrected P-value.
[a] Model which include only the variable significant in the final analysis. [b] Model which include all variables significant in multivariate analysis (Table 3 in the paper). * Significant p-values (< 0.05).

Table S5. Multivariate Cox regression analysis of BCR-free survival in relation to known preoperative D'Amico risk classification in our PC cohort (n=194);.

Variable	D'Amico alone			D'Amico + *DOCK2*		
	HR (95% CI)	P-value	C-index[a]	HR (95% CI)	P-value	C-index[b]
DOCK2 - continuous	-	-	-	2.26 (1.40-3.63)	0.001*	0,645[b]
D'Amico low vs inter	2.72 (1.07-6.92)	0.036*	0,599[a]	2.26 (0.88-5.79)	0.089	
D'Amico low vs high	4.30 (1.72-10.73)	0.002*		3.49 (1.39-8.76)	0.008*	

HR, Hazard ratio; CI, confidence interval; low, D'Amico low risk patients (n=23); inter, D'Amico intermediate risk patients (n=80); high, D'Amico high risk patients (n=91); [a] Model which include only D'Amico risk classification; [b] Model which include both *DOCK2* and D'Amico risk classification; * Significant p-value < 0.005.

Table S6. Primer and probe sequences for qMSP assay used for small scale validation (Figure S2 and Table S1). Primer and probe sequences for LNA-based assays are available upon request.

Primer	Forward primer sequence 5′ to 3′	Reverse primer sequence 5′ to 3′
cg12799885	AGGTCGTATTTTCGTTGTGG	CTTAACTAATTTCGCCGCGA
DOCK2	GTAGGTAGCGCGTTTTGT	ACGAAACAAATAACGAAAAA
FBXO30	TCGGACGGGATAGTTTATT	CCCTCCCCAACTAATTACC
GRASP	GTTGGGTGTTTCGATTTTTC	AAAAACCACCGAACCTAAAC
HIF3A	GCGAGTTAAGAACGTTTCG	CACCCCTACAATCCCTAAAA
MOB3B	GTTTTCGGGGTTGGAGTTTT	AACGACCCTTATTCCTACCG
PFKP	TTTTTAGTGGTTCGTAAGATTTTT	ACCTAAAAACGAAAATCGAAA
TPM4	GGCGTATCGTGTAGGTTTC	ACGTTTTCATCCTCATTCTCC

Probe	Probe sequence 5′ to 3′	Modification
cg12799885	CGAGAGCGAGTTGAGTGGGGG	5′6-FAM, 3′BHQ-1
DOCK2	TTTCGCGTCGTCGGTTTCGT	5′6-FAM, 3′BHQ-1
FBXO30	GGTCGAGGCGTTATTTCGCG	5′6-FAM, 3′BHQ-1
GRASP	CGTTGTTGCGAAGGTCGTGG	5′6-FAM, 3′BHQ-1
HIF3A	GGGCGCGTAGTTGGAGGTATATTT	5′6-FAM, 3′BHQ-1
MOB3B	TCGGCGTTTGGGTTTTTGGTCGG	5′6-FAM, 3′BHQ-1
PFKP	GGGAGTTTCGGGGTTCGGTT	5′6-FAM, 3′BHQ-1
TPM4	GGGGTTGGCGTCGGGGTTTC	5′6-FAM, 3′BHQ-1

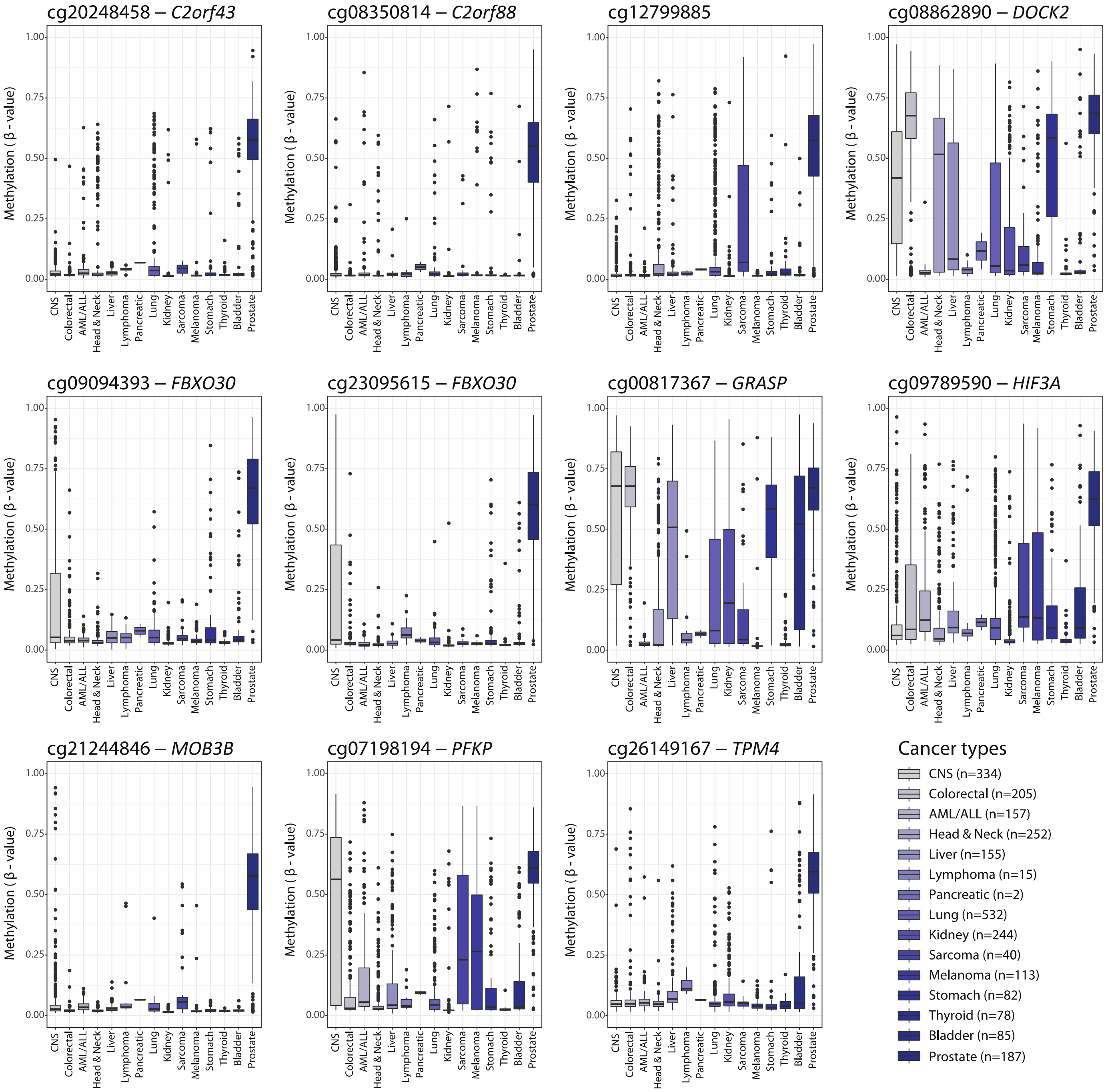

Figure S1. Cancer type related methylation levels (ß-value) of the 11 selected biomarker candidates. The coloured boxes indicate 25 -75th percentile, with the black line indicating the median. Top whiskers are 3th quartile + 1.5 interquartile range, bottom whiskers are 1st quartile – 1.5 interquartile range. Dots indicates outliers.

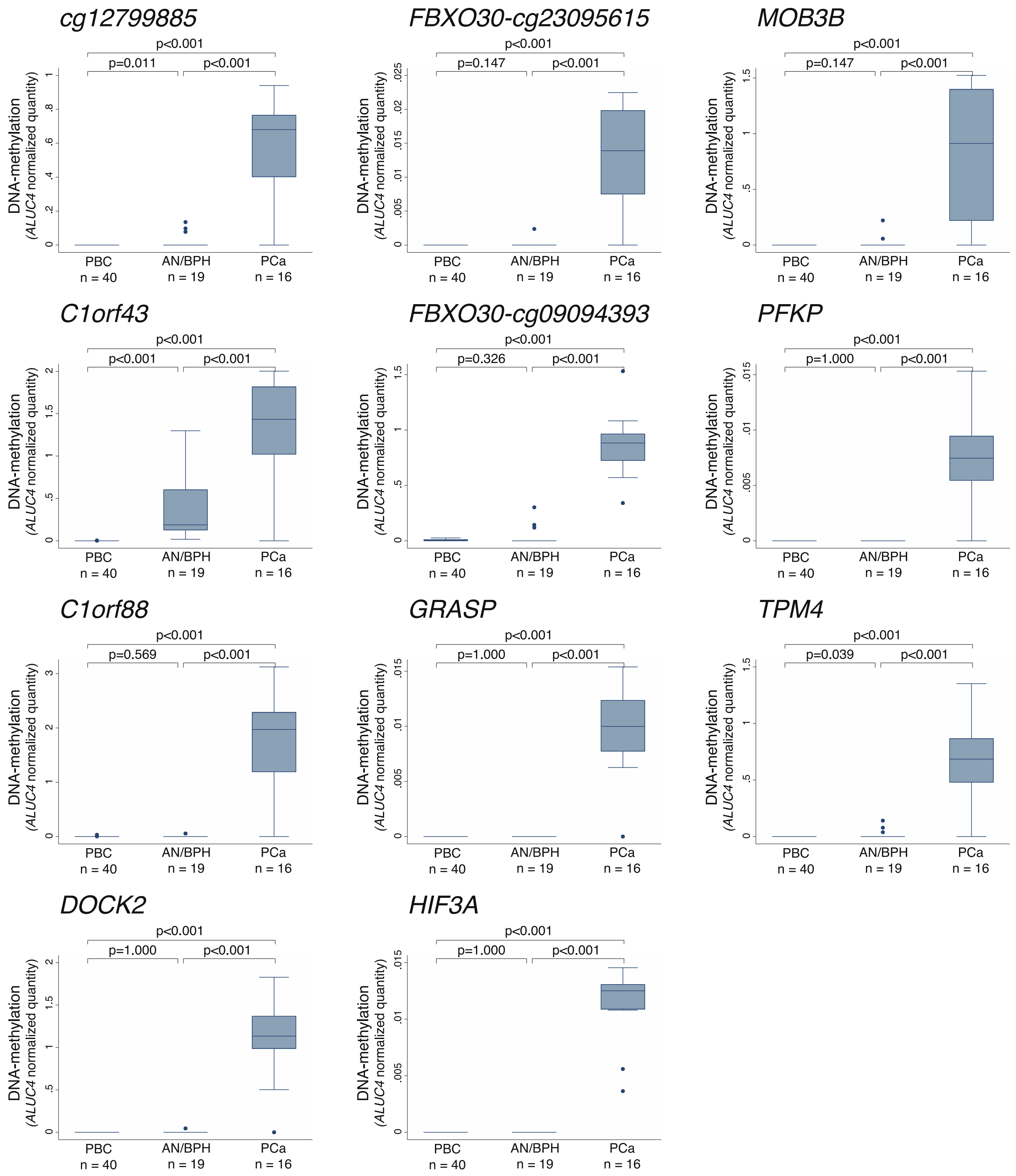

Figure S2. Boxplot over *ALUC4* normalized methylation levels (qMSP) for eleven biomarker candidates selected for small scale validation. PCa: Prostate cancer tissue samples, AN: adjacent normal samples, BPH: benign prostate hyperplasia samples, PBC: peripheral blood cell samples (buffy coat). The coloured boxes indicate 25 -75th percentiles, with the black line indicating the median. Top whiskers are 3th quartile + 1.5 interquartile range, bottom whiskers are 1st quartile – 1.5 interquartile range. Dots indicates outliers. P-value from Wilcoxon Mann-Whitney test.

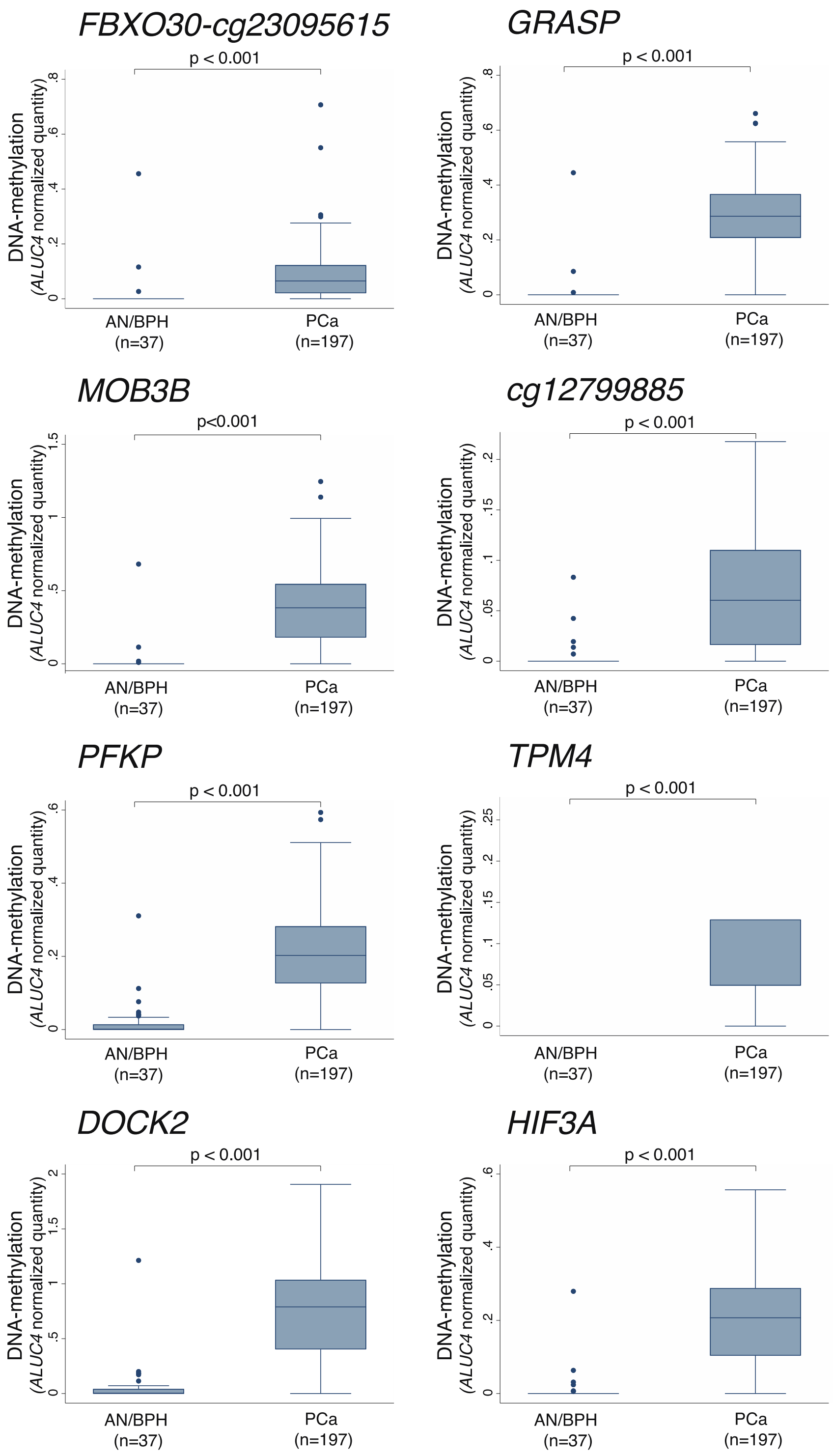

Figure S3. Boxplot over ALUC4 normalized methylation level (qMSP) for the eight biomarker included in large scale validation. PCa: Prostate cancer tissue, AN: adjacent normal, BPH: benign prostate hyperplasia. The coloured boxes indicate 25 -75th percentiles, with the black line indicating the median. Top whiskers are 3th quartile + 1.5 interquartile range, bottom whiskers are 1st quartile – 1.5 interquartile range. Dots indicates outliers (> 3th quartile + 1.5 interquartile range). P-value from Wilcoxon Mann-Whitney test.

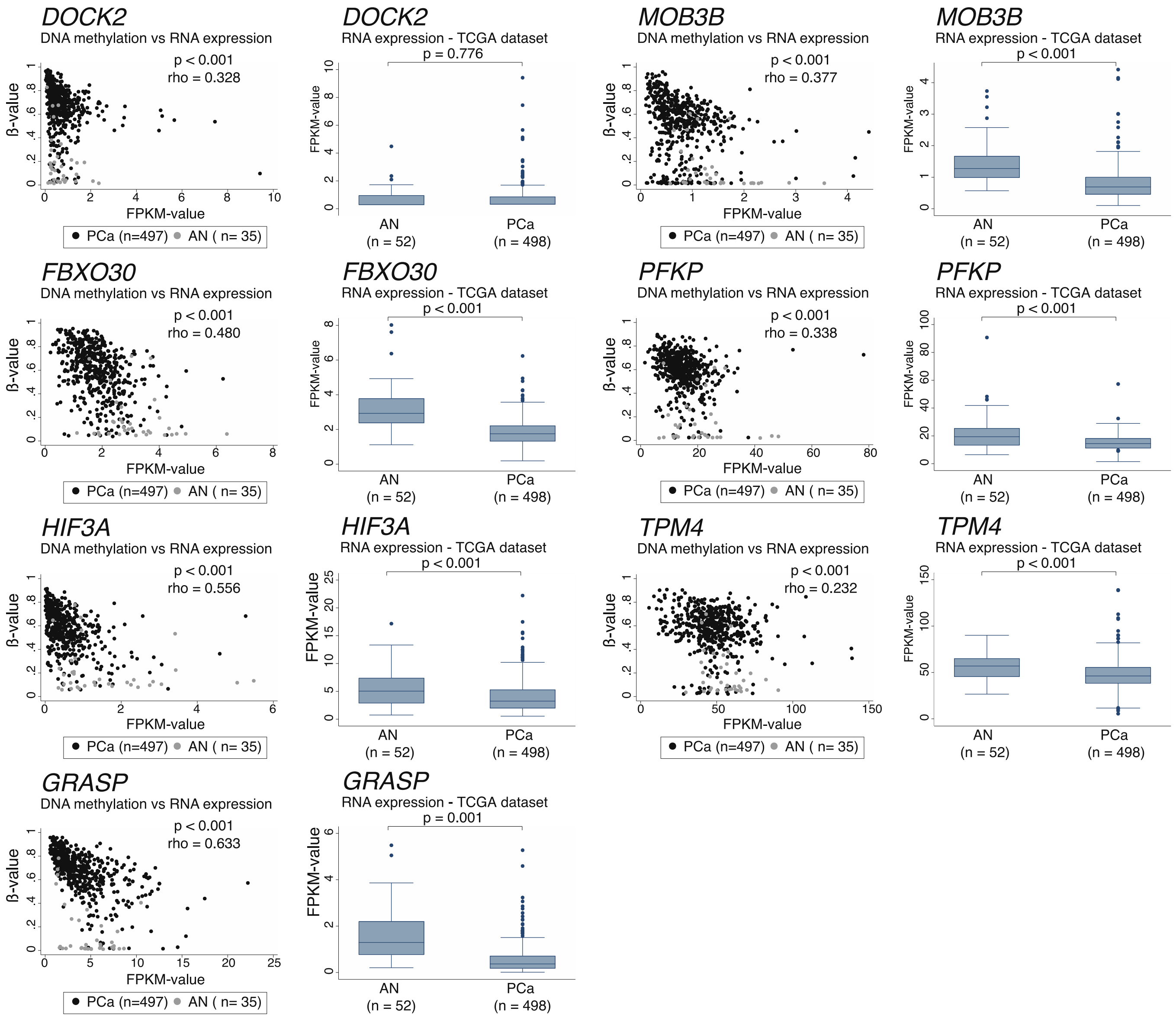

Figure S4. Correlation between RNA expression and DNA methylation in the TCGA cohort. Box plots over RNA expression. PCa: Prostate cancer tissue, AN: adjacent normal.
The coloured boxes indicate 25 -75th percentiles, with the black line indicating the median. Top whiskers are 3th quartile + 1.5 interquartile range, bottom whiskers are 1st quartile – 1.5 interquartile range. Dots indicates outliers (> 3th quartile + 1.5 interquartile range). P-value from Wilcoxon Mann-Whitney test.
